# Molecular Landscape and Prognostic Biomarker Analysis of Advanced Pancreatic Cancer and Predictors of Treatment Efficacy of AG Chemotherapy

**DOI:** 10.3389/fonc.2022.844527

**Published:** 2022-05-18

**Authors:** Juan Du, Xin Qiu, Changchang Lu, Yahui Zhu, Weiwei Kong, Mian Xu, Xin Zhang, Min Tang, Jun Chen, Qi Li, Aimei Li, Jian He, Qing Gu, Lei Wang, Yudong Qiu, Baorui Liu

**Affiliations:** ^1^ The Comprehensive Cancer Center of Drum Tower Hospital, Medical School of Nanjing University & Clinical Cancer Institute of Nanjing University, Nanjing, China; ^2^ The Comprehensive Cancer Center of Drum Tower Hospital, Clinical College of Traditional Chinese and Western Medicine, Nanjing University of Chinese Medicine, Nanjing, China; ^3^ Shanghai OrigiMed Co, Ltd, Shanghai, China; ^4^ Department of Radiology, Nanjing Drum Tower Hospital, The Affiliated Drum Tower Hospital of Nanjing University Medical School, Nanjing, China; ^5^ Department of Pathology, Nanjing Drum Tower Hospital, The Affiliated Drum Tower Hospital of Nanjing University Medical School, Nanjing, China; ^6^ Department of Nuclear Medicine, The Affiliated Drum Tower Hospital of Nanjing University Medical School, Nanjing, China; ^7^ State Key Lab of Novel Software Technology, Nanjing University, Nanjing, China; ^8^ Department of Gastroenterology, The Affiliated Drum Tower Hospital of Nanjing University Medical School, Nanjing, China; ^9^ Department of Hepatopancreatobiliary Surgery, The Affiliated Drum Tower Hospital of Nanjing University Medical School, Nanjing, China

**Keywords:** mutational analysis, advanced pancreatic cancer, metastasis, prognostic biomarker, AG chemotherapy

## Abstract

**Purpose:**

Although mutational analysis of pancreatic cancer has provided valuable clinical information, it has not significantly changed treatment prospects. The purpose of this study is to further investigate molecular alterations in locally advanced pancreatic cancer and identify predictors of the efficacy of nab-paclitaxel plus gemcitabine (AG) chemotherapy.

**Experimental design:**

Tumor samples from 118 pancreatic cancer patients who received AG chemotherapy as first-line treatment were sequenced and genomic profile was generated. Molecular alterations and the involved signaling pathways were analyzed. Genes with a significant difference in mutation frequency between primary and metastatic tumors were identified, and prognostic-related mutant genes were screened using SPSS version 22.0.

**Results:**

The most common altered genes in the patients were *KRAS* (94.9%), *TP53* (81.4%), *CDKN2A* (36.4%), and *SMAD4* (22.9%). The mutational frequencies of *CDKN2B* (14.8% vs. 0%, p = 0.001)*, FAT3* (7.4% vs. 0%, p = 0.041)*, MTAP* (13% vs. 1.6%, p = 0.023), and *SMAD4* (31.4% vs. 15.6%, p = 0.049) in metastatic tumors were significantly higher than that in primary tumors. *TP35* and *KRAS* mutations were significantly correlated with objective response rate, while *EPHA7, RNF43*, and *HMGA2* mutations were significantly correlated with disease control rate. Additionally, patients with *TGFR2B, FGF23, EPHA7, SMARCA4, CARD11, ADGRA2, CCNE1*, and *ACVR2A* alterations had a worse overall survival. Further, *EPHA7, CARD11, NOTCH1, GATA6, ACVR2A*, and *HMGA2* mutations indicated undesirable progression-free survival.

**Conclusions:**

*CDKN2B*, *FAT3*, *MTAP*, and *SMAD4* may be biomarkers that distinguish primary tumors from metastases. *EPHA7* mutation may serve as a prognostic biomarker to predict the treatment efficacy of AG chemotherapy in locally advanced pancreatic cancer.

## Introduction

Pancreatic cancer is a highly invasive malignant tumor and has the worst prognosis of all human cancers, with a 5 year survival rate of 11% (1). Despite great advances in treatments like radiotherapy and chemotherapy and improvements in surgical techniques, pancreatic cancer will likely become the second leading cause of cancer-related deaths by 2030 (2). So far, surgical resection is the only curative treatment. Unfortunately, only approximately 15–20% of pancreatic cancer patients have resectable disease at diagnosis (3). Even worse, among these patients the median survival is about 8.5 to 11.1 months owing to early metastasis (4, 5). Indeed, aggressive and cytotoxic chemotherapy is still the standard treatment for pancreatic cancer. The National Comprehensive Cancer Network (NCCN) lists nab-paclitaxel plus gemcitabine (AG chemotherapy) as the preferred first-line therapy for the treatment of locally advanced pancreatic cancer and metastatic pancreatic cancer. However, the median progression-free survival of metastatic pancreatic cancer treated with AG chemotherapy was only 5.5 months, and the median overall survival of metastatic pancreatic cancer was 8.5 months (6). Moreover, due to the absence of effective prognostic and predictive biomarkers and the limited clinical success of preferred therapies, the successful treatment of pancreatic cancer is still elusive (7). Hence, to improve existing treatments and develop novel therapeutic strategies, a better understanding of the molecular pathology of pancreatic cancer is vital.

Next-generation sequencing (NGS) is now widely available for examination of tumor samples in commercial laboratories, and advances in NGS-based genomics have greatly increased our understanding of the molecular basis of pancreatic cancer. Based on comprehensive integrated genomic and transcriptomic analysis, a study in 2016 classified pancreatic cancer into four subtypes (stable, locally rearranged, scattered, and unstable) that are associated with specific histopathological characteristics (3). Genomic analyses revealed four commonly mutated genes (*KRAS, TP53, SMAD4*, and *CDKN2A*) in pancreatic cancer. These mutations cluster in core molecular pathways, including DNA damage repair, cell cycle regulation, TGF-β signaling, chromatin regulation, and axonal guidance (8, 9). Further, several molecular profiling studies revealed that up to 48% of pancreatic cancers harbor therapeutically relevant genomic alterations, such as *ERBB2, MET, FGFR1, CDK6, PIK3R3*, and *PIK3CA* at low individual patient prevalence (3, 8, 10). In addition, a recent study showed that tumor fraction was remarkably higher in metastatic than localized tumors, and genetic heterogeneity was found between distinct metastatic tumors, especially in different organs (11). However, our knowledge of the genomic landscape of metastatic pancreatic cancer remains limited.

In this study we performed genomic analysis of 118 pancreatic cancer patients using NGS. Primary tumors were obtained from 64 patients and metastatic tumors from 54 patients, and the mutational landscapes and mutated-gene related pathways of the primary and metastatic tumors were mapped and compared. Furthermore, mutated genes associated with prognosis were screened for the potential prediction of treatment response and development of new targeted treatments.

## Materials and Methods

### Patients

A total of 118 pancreatic cancer patients from the Nanjing Drum Tower Hospital, the Affiliated Hospital of Nanjing University Medical School, were enrolled for this study from 2018 to 2020. Tumor samples or biopsy tissues were collected from these patients for the detection of genomic alterations. Pancreatic cancer patients with locally advanced tumors or metastases were included. Relevant basic information, family history, clinical and pathological features, and treatment history were collected. All of the patients received AG chemotherapy as a first-line treatment, and some received second-line (and third-line therapy) according their own will. Treatment response was assessed with abdominal enhanced computed tomography and magnetic resonance imaging following the criteria of Response Evaluation Criteria in Solid Tumors (RECIST). Treatment response was quantitatively classified into four categories: complete response (CR), partial response (PR), stable disease (SD), and progressive disease (PD). The information for the 118 patients has not been previously published.

### Ethics

The project was approved by the Ethics Committee of Nanjing Drum Tower Hospital (approval number: EXPLORE-PC101). Informed consent was provided by all patients or their immediate families. This work was conducted in accordance with the guidelines of the Declaration of Helsinki. All methods used in this protocol were carried out in accordance with the relevant guidelines and regulations.

### Identification of Genomic Alterations and Tumor Mutational Burden

The genomic alterations were identified using the NGS-based YuanSuTM450 gene panel (OrigiMed, Shanghai, China), which covers all the coding exons of 450 cancer-related genes that are frequently rearranged in solid tumors. The genes were captured and sequenced with a mean depth of 800× using Illumina NextSeq 500. The procedures followed those described by Frampton et al. (12). GAs) were identified as previously described methods (13): single nucleotide variants (SNV) were identified using MuTect (v1.7). Insertion-deletions (indels) were identified using PINDEL (V0.2.5). The functional impacts of GAs were annotated using SnpEff3.0. Copy number variation (CNV) regions were identified using Control-FREEC (v9.7) with the following parameters: window = 50,000 and step = 10,000. Gene fusions were detected through an in-house developed pipeline. Gene rearrangements were assessed using Integrative Genomics Viewer (IGV). TMB was estimated by counting the coding somatic mutations, including SNV and indels, per megabase of the sequence examined in each patient. The TMB value was further divided into two groups: high TMB (TMB-H), defined as ≥10 mutations/Mb, and low TMB (TMB-L), defined as <10 mutations/Mb.

### Statistical Analysis

Statistical analyses were performed using SPSS version 22.0 (SPSS Inc., Chicago, IL, USA). Overall survival (OS) was defined as the time from the start of AG therapy to death from any cause. Progression-free survival (PFS) was defined as the time from the start of AG therapy to disease progression. The objective response rate (ORR) was defined as the percentage of patients that achieved PR and SD. The disease control rate (DCR) was defined as the percentage of patients that achieved PR. Fisher’s exact test was used to analyze associations between categorical variables. The Student’s t−test and the Wilcoxon rank test were used to analyze associations between normally distributed data and non-normally distributed data, respectively. The Kruskal test was used to analyze associations between multiple groups of nonparametric data. A value of p < 0.05 was considered statistically significant.

## Results

### Patient Characteristics

A total of 118 pancreatic cancer patients were enrolled in this study. The median age of these patients was 61 years old (range = 33−85 years old); 67 of them were male (56.8%) and 51 were female (43.2%). The tumor tissues were divided into primary tumors (n = 64, 54.2%) and metastatic tumors (n = 54, 45.8%). Based on tumor stage, there were 36 patients at stage III (30.5%) and 82 patients at stage IV (69.5%). The median TMB of the 118 patients was 3.1 Mut/Mb (range = 0.0−69.5). In the cohort, 4 patients were identified as TMB-H (3.4%) while 114 patients were identified as TMB-L (96.6%). Microsatellite stability (MSS) status was also measured in all patients; 1 patient had high microsatellite instability (MSI-H), and 117 patients had MSS. Detailed clinical characteristics of each patient are listed in **Table 1**.

**Table 1:** Clinical characteristics of patients.

Characteristics	n=118
Age, years (median, IQR)	61 (33-85)
Gender, n (%)	
Male	67 (56.8%)
Female	51 (43.2%)
Source of samples, n (%)	
Primary	64 (54.2%)
Metastasis	54 (45.8%)
Stage	
III	36 (30.5%)
IV	82 (69.5%)
TMB (median, IQR)	3.1 (0.0-69.5)
TMB status, n (%)	
TMB≥10	4 (3.4%)
TMB<10	114 (96.6%)
MSI status, n (%)	
MSS	117 (99.0%)
MSI-H	1 (1.0%)

### Significant Genomic Alterations in Pancreatic Cancer

**Figure 1:**
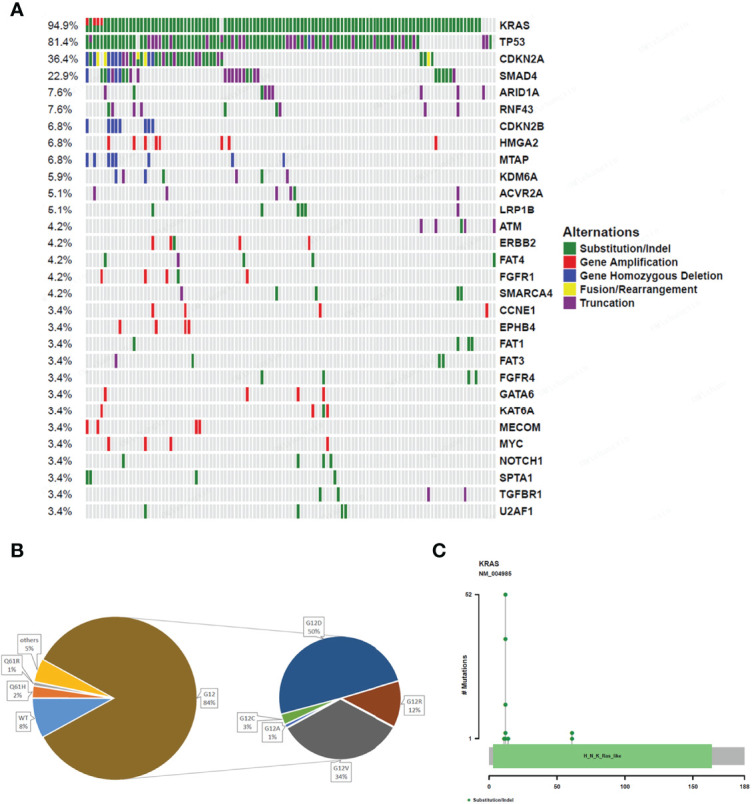
Gene alterations of 118 pancreatic cancer patients. **(A)** The mutation profiles. The panel shows the matrix of mutations with each type of mutation in a different color. Each column denotes an individual tumor and each row represents a gene. The right panel shows the name of mutations and the left panel shows the proportion of mutations. Green; Substitution/Indel, Red; Gene amplification, Blue; Gene homozygous deletion, Yellow; Fusion/Rearrangement, Purple; Truncation. **(B)** Ratio of different mutation types in *KRAS* gene. **(C)** The distribution map of *KRAS* gene mutation sites showing the distribution of mutation sites.

A total of 792 variations in 267 genes, including 447 (56.4%) substitution/indels, 157 (19.8%) gene amplification, 135 (17.0%) truncation, 18 (2.3%) fusions/rearrangements, 35 (4.4%) gene homozygous deletions, and 23 (2.9%) gene homozygous deletions, were detected in the 118 pancreatic cancer patients. The most common gene alterations in the study group were *KRAS* (94.9%), *TP53* (81.4%), *CDKN2A* (36.4%), and *SMAD4* (22.9%) (**Figure 1A**). Notably, the most common *KRAS* and *TP53* variants observed were substitution/indels (**Figure 1**). The most frequent mutations were in codon 12 of *KRAS* (84%), including *KRAS* G12D (50%) and G12V (34%) (**Figures 1B, C**).

### Mutated Gene-Related Pathways in Pancreatic Cancer

**Figure 2:**
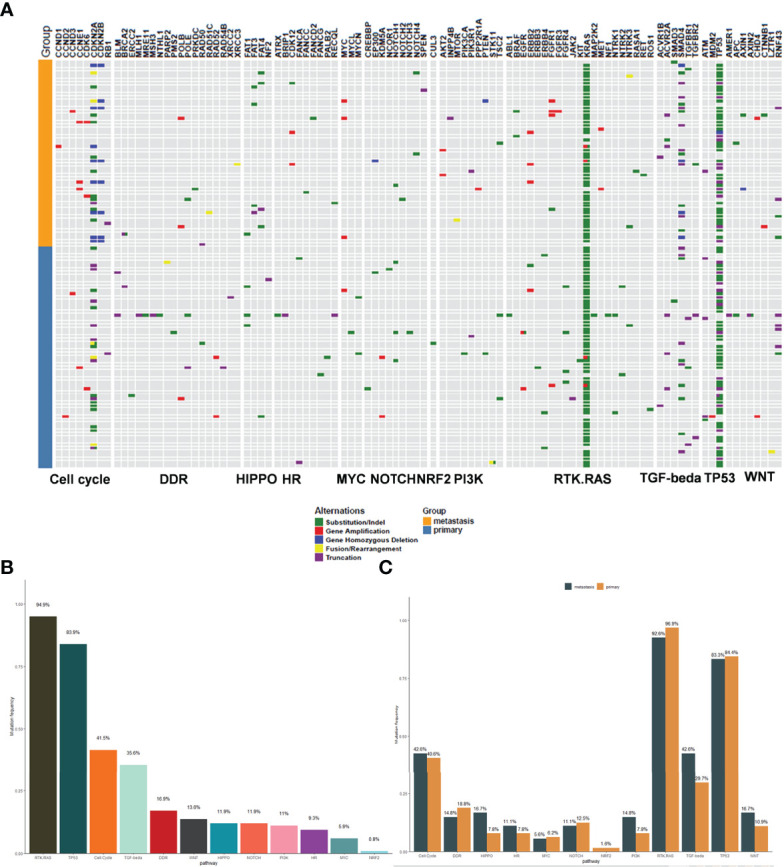
The signaling pathway involved in the mutant genes. **(A)** The map of mutant genes and the involved signal pathways. Light blue represents the primary tumors and orange represents the metastatic tumors. The upper panel shows the mutant genes and the lower panel shows the signal pathways. Green; Substitution/Indel, Red; Gene amplification, Blue; Gene homozygous deletion, Yellow; Fusion/Rearrangement, Purple; Truncation. **(B)** The mutation frequency of the signaling pathways involved in the mutant genes. **(C)** The comparison of mutation frequency of the signaling pathways between primary and metastatic tumors.

In order to identify the functions of the mutated genes and understand how these mutations contribute to selective advantages during tumorigenesis, their involved regulatory pathways were analyzed. All the mutated genes were mainly involved in the following signaling pathways: RTK.RAS (94.9%), TP53 (83.9%), cell cycle (41.5%), TGF-β (35.6%), DDR (16.9%), WNT (13.6%), HIPPO (11.9%), NOTCH (11.9%), PI3K (11%), HR (9.3%), MYC (5.9%), and NFR2 (0.8%) (**Figures 2A, B**). RTK.RAS pathway alterations were present in most patients, predominantly due to *KRAS* mutations (94%). In patients lacking *KRAS* mutations, other RTK.RAS genes were often mutated, including *BRAF, EGFR, ERBB2/3/4, FGFR1/3/4*, and *ROS1* (**Figure 2A**). There was no significant difference in the involved regulatory pathways between primary and metastatic tumors (**Figure 2C**).

### Significant Genomic Alterations in Primary and Metastatic Pancreatic Cancer

**Figure 3:**
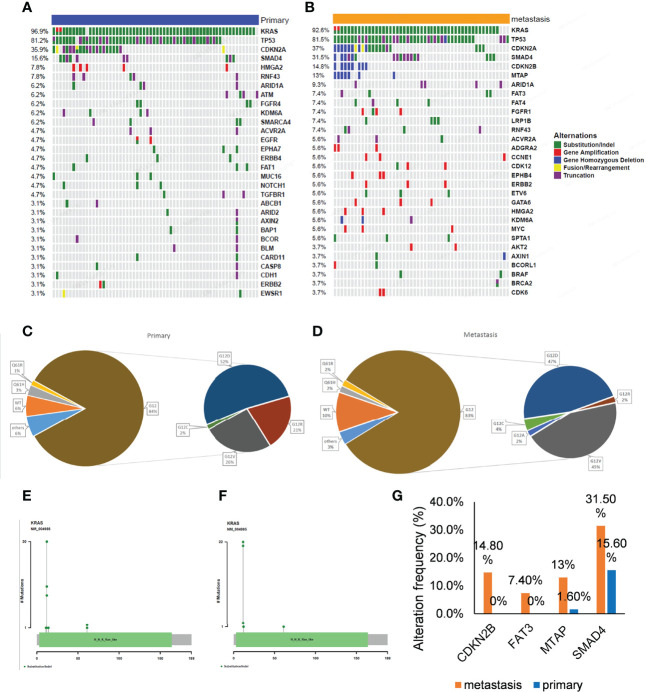
Gene alterations of 64 primary and 54 metastatic tumors. **(A, B)** The mutation profiles. The panel shows the matrix of mutations with each type of mutation in a different color. Each column denotes an individual tumor and each row represents a gene. The right panel shows the name of mutations and the left panel shows the proportion of mutations. Green; Substitution/Indel, Red; Gene amplification, Blue; Gene homozygous deletion, Yellow; Fusion/Rearrangement, Purple; Truncation. **(C, D)** Ratio of different mutation types in *KRAS* gene. **(E, F)** The distribution map of *KRAS* gene mutation sites showed the distribution of mutation sites. **(G)** Genes with a significant difference in mutation frequency between primary and metastatic tumors.

Tumor metastasis of pancreatic cancers is an important prognostic factor. To explore the molecular mechanisms involved, we performed genomic analysis of the primary and metastatic tumors in this study and analyzed their mutational characteristics individually. The most common gene alterations in the 64 primary pancreatic cancer were *KRAS* (96.9%), *TP53* (81.2%), *CDKN2A* (35.9%), and *SMAD4* (15.6%) (**Figure 3A**). The most common gene alterations in the 54 metastatic pancreatic cancer were *KRAS* (92.6%), *TP53* (81.5%), *CDKN2A* (37%), *SMAD4* (31.5%), *CDKN2B* (14.8%), and *MTAP* (13%) (**Figure 3B**). Similarly, the most common changes in *KRAS* and *TP53* were substitution/indels in primary and metastatic tumors (**Figures 3A, B**). *KRAS* G12D and G12V showed the highest variation frequency (43% and 22% in primary tumors, and 47% and 45% in metastatic tumors, respectively) (**Figures 3C–F**). To further explore the association and compare the molecular differences between primary and metastatic tumors, the frequencies of the most common mutations were compared. As shown in **Figure 3G**, the mutational frequencies (metastatic vs. primary) of *CDKN2B* (14.8% vs. 0%, p = 0.001)*, FAT3* (7.4% vs. 0%, p = 0.041)*, MTAP* (13% vs. 1.6%, p = 0.023), and *SMAD4* (31.4% vs. 15.6%, p = 0.049) in metastatic tumors were significantly higher than in primary tumors. Detailed numbers and mutational frequencies are listed in **Table 2**. Notably, *CDKN2B* and *FAT3* alterations occurred only in patients with metastatic tumors.

**Table 2:** Detailed patient number and the mutational frequency.

	primary (n=64)	metastasis (n=54)	Total	P-value
*SMAD4*	10 (15.6%)	17 (31.5%)	27 (22.9%)	0.049
*MTAP*	1 (1.6%)	7 (13.0%)	8 (6.8%)	0.023
*CDKN2B*	0 (0%)	8 (14.8%)	8 (6.8%)	0.001
*FAT3*	0 (0%)	4 (7.4%)	4 (3.4%)	0.041

### Potential Prognostic Biomarker and Predictor of Treatment Efficacy of AG Chemotherapy Analysis

**Figure 4:**
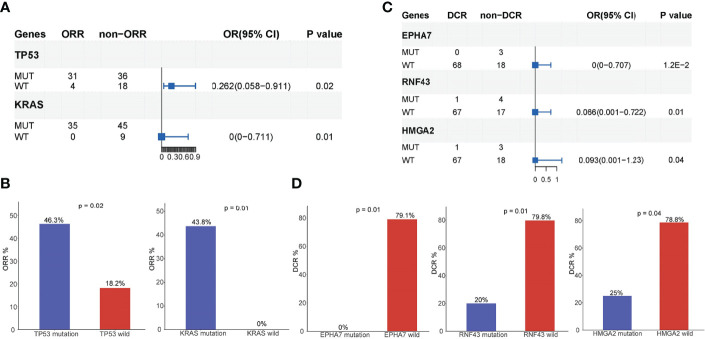
Analysis of mutated genes associated with prognosis. **(A, B)** *TP35* and *KRAS* mutations were significantly correlated to ORR of AG chemotherapy. The ORR of *TP53* mutant patients is 46.3%, while the ORR of *TP53* wild type patients is 18.2%.The ORR of *KRAS* mutant patients is 43.8%, while the ORR of *KRAS* wild type patients is 0%. **(C, D)** *EPHA7, RNF43*, and *HMGA2* mutations were significantly correlated to the DCR of AG chemotherapy. The DCR of *EPHA7* mutant patients is 0%, while the DCR of *EPHA7* wild type patients is 79.1%.

**Figure 5:**
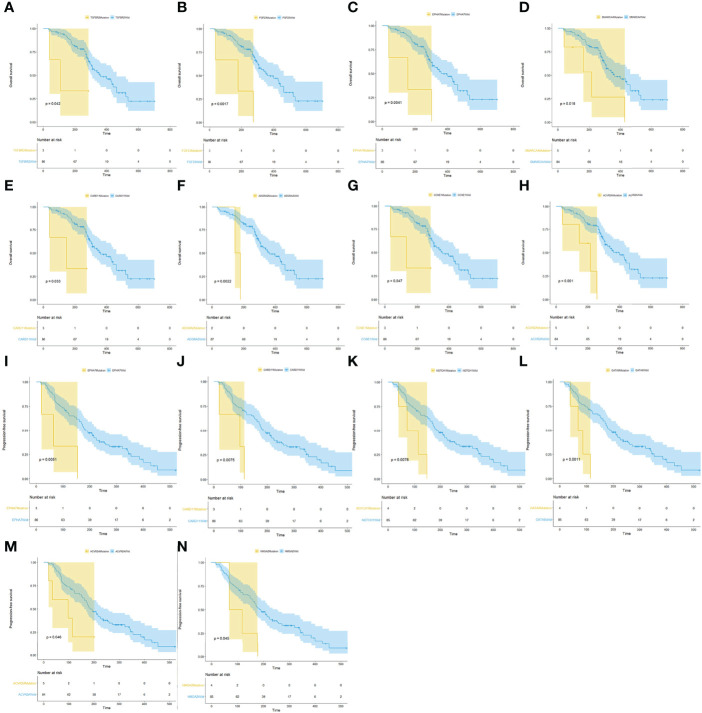
Analysis of mutated genes associated with prognosis. **(A–H)** Survival analysis showed that patients with *TGFR2B, FGF23, EPHA7, SMARCA4, CARD11, ADGRA2, CCNE1*, and *ACVR2A* alterations had a worse OS. **(I–N)** *EPHA7, CARD11, NOTCH1, GATA6, ACVR2A*, and *HMGA2 S* mutations indicated undesirable PFS.

Of the 118 patients, 89 had a complete follow-up with the best objective response assessment and PFS and OS evaluations. Hence, the mutational and follow-up information of these 89 patients was used to analyze the influence of mutated genes on clinical response rate and prognosis. The results revealed that the ORR of *TP53* mutated patients (46.3%) was significantly higher than that of *TP53* wild type patients (18.2%) and that the ORR of *KRAS* mutated patients (43.8%) was significantly higher than that of *KRAS* wild type patients (0%), demonstrating that *TP35* (p = 0.02) and *KRAS* (p = 0.01) mutations were significantly correlated to better ORR (**Figures 4A, B**). At the same time, the DCR of *EPHA7* mutated patients (0%), *ERNF43* mutated patients (20%), and *HMGA2* mutation patients (25%) was dramatically lower than that of *EPHA7* wild type patients (79.1%), *ERNF43* wild type patients (79.8%), and *HMGA2* wild type patients (78.8%) (**Figures 4C, D**), verifying that *EPHA7* (p = 0.01)*, RNF43* (p = 0.01), and *HMGA2* (p = 0.04) mutations were significantly correlated to worse DCR. In addition, patients with *TGFR2B, FGF23, EPHA7, SMARCA4, CARD11, ADGRA2, CCNE1*, and *ACVR2A* alterations had a worse OS (**Figures 5A–H**), and *EPHA7, CARD11, NOTCH1, GATA6, ACVR2A*, and *HMGA2* mutations indicated undesirable PFS (**Figures 5I–N**).

## Discussion

Pancreatic cancer remains one of the most fatal malignancies with a very poor prognosis. In fact, the primary clinical treatment for advanced and/or metastatic pancreatic cancer remains conventional chemotherapy, such as AG chemotherapy (5, 6) and the FOLFIRINOX regimen, but the treatment results are unsatisfactory. Currently, the NCCN guidelines for pancreatic cancer have no guidance for individualized chemotherapy, save for a recommendation that patients with germline *BRCA1/2* gene mutations choose platinum chemotherapy. Hence, it is essential to be able to select populations that aremore likely to respond to existing treatments, such as AG chemotherapy, and to develop novel targeted therapeutic strategies for pancreatic cancer patients.

In recent years, many researchers have examined the pancreatic cancer genome using NGS to lay down the molecular foundation for targeted therapy. Waddell et al. revealed that *KRAS* and *CDKN2A* were mutated in over 90% of pancreatic cancers, while the *TP53* and *SMAD4* genes were mutated in 75% and 55% of the patients, respectively (8). In addition, Kamisawa et al. showed that pancreatic cancer cells were characterized by a hypermutated landscape, with the *KRAS* oncogene, *TP53, CDKN2A*, and *SMAD4* tumor suppressor genes the four most commonly mutated genes (14). Similarly, our results showed that the most common gene alterations in the 118 pancreatic cancer patients studied were in *KRAS* (94.9%), *TP53* (81.4%), *CDKN2A* (36.4%), and *SMAD4* (22.9%). In addition, *KRAS* mutations in codon 12 were the most common (84%), with *KRAS* G12D and G12V the highest variation frequency (50% and 34%, respectively). These results are in agreement with the study done by Shinichi et al., which showed activating mutations in *KRAS* in 92% of pancreatic cancer patients, with mutations in codon 12 being the most common (90%) (15).

Metastasis is a vital risk factor for poor prognosis in pancreatic cancer patients because patients with metastases are usually not suitable candidates for operative treatment. However, the specific molecular mechanism of pancreatic cancer metastasis is not clear and may involve the abnormal expression of cancer-related genes and the disorder of related signaling pathways. A study of 2552 pancreatic ductal adenocarcinoma (PDAC) patients found that the most common gene alterations were mutations in *KRAS* and *PTEN* (59% and 62%, respectively), with differences in prevalence by site of metastasis (p = 0.042 and p = 0.037, respectively). *KRAS* mutations were more commonly found in metastases in the lung (72%) than other sites (59%, p = 0.042). Low expression of *ERCC1* was found in 49% of lung metastases from PDAC but only 15% in PDAC in the pancreas (p < 0.001) (16). In another study, 109 differentially expressed genes related with pancreatic cancer metastasis were screened by analyzing the pancreatic cancer metastasis-related gene chip data in the GEO database. Of these, 49 were upregulated and 60 were downregulated (17).

In our study, the most common gene alterations in the 66 primary pancreatic cancers were *KRAS* (96.9%), *TP53* (81.2%), *CDKN2A* (35.9%), and *SMAD4* (15.6%) (**Figure 2A**). Further, the most common gene alterations in the 54 metastatic pancreatic cancers were *KRAS* (92.6%), *TP53* (81.5%), *CDKN2A* (37%), *SMAD4* (31.5%), *CDKN2B* (14.8%), and *MTAP* (13%) (**Figure 2B**). Importantly, the mutational frequency of *CDKN2B* (14.8% vs. 0%, p = 0.001), *FAT3* (7.4% vs. 0%, p = 0.041), *MTAP* (13% vs. 1.6%, p = 0.023), and *SMAD4* (31.4% vs. 15.6%, p = 0.049) in metastatic tumors were significantly higher than those in primary tumors. Notably, *CDKN2B* and *FAT3* alterations occurred only in metastatic tumors. Other studies have shown that loss of SMAD4 expression is associated with metastasis in pancreatic cancer (18–20). Evidently, the relationship between metastatic pancreatic cancer and other significantly mutated genes, such as *CDKN2B*, *FAT3*, and *MTAP*, remains obscure.

All detected mutations are involved in the dysregulation of core signaling pathways that affect not only the specific features of tumor cells, such as proliferation and migration, but also the tumor microenvironment (14). Approximately 32 recurrently mutated genes in pancreatic cancer that aggregate into 10 core pathways have been reported: KRAS, TGF-β, WNT, NOTCH, ROBO/SLIT signaling, G1/S transition, SWI-SNF, chromatin modification, DNA repair, and RNA processing (3). Another study revealed that MAPK (87%), PI3K/AKT/mTOR (19%), DNA repair (15%), cell cycle (11%), and AKT/mTOR (19%) pathway alterations were present in pancreatic cancer patients (21). In this study, all the mutated genes were mainly involved in the following signaling pathways: RTK.RAS, TP53, cell cycle, TGF-β, DDR, WNT, HIPPO, NOTCH, PI3K, HR, MYC, and NFR2. We found RTK.RAS (94.9%) pathway alterations in most pancreatic cancer patients. These were predominantly due to *KRAS* mutations, but in patients lacking *KRAS* mutations, other RTK.RAS genes were often mutated, such as *BRAF, EGFR, ERBB2/3/4, FGFR1/3/4*, and *ROS1*. This RTK.RAS signaling pathways dysregulation is consistent with previous studies (3, 8, 9). Additionally, 11.9% of patients had some molecular defect in the PI3K pathway, including mutations in *STK11, PIK3CA, TSC2*, and *AKT2*. These frequencies were comparable to those from other published studies (21, 22). Furthermore, in a retrospective analysis, 13.9% (94/677) of patients with actionable mutations had mutations in the DDR pathway (23–26). Similarly, our data showed that 16.9% of pancreatic cancer patients had DDR pathway alterations, with actionable mutations such as *BRCA2*, and *POLE.* However, our study showed no significant difference in the regulatory pathways affected in primary and metastatic tumors.

Up to 85% of pancreatic cancer patients are diagnosed with already locally advanced and/or metastatic tumors because of a lack of specific symptoms and early biomarkers for this highly aggressive disease (14). Therefore, it is particularly important to explore molecular biomarkers for early prediction and prognosis. An activating point mutation of the *KRAS* oncogene in codon 12 (exon 2) is the initiating event in the majority of PDAC patients (27, 28). The point mutation of *KRAS* damages the intrinsic GTPase activity of RAS and prevents GAPs from promoting the conversion of GTP (active) to GDP (inactive). Thus, KRAS is permanently bound to GTP and activates downstream signaling pathways, increasing cellular proliferation and survival (28, 29). Several studies have investigated whether *KRAS* mutations influence the prognosis of PDAC, and the results showed that the *KRAS* mutation has a negative influence on the prognosis of PDAC patients, especially G12D, G12R, or a combination of the two mutants (30, 31). Our study results are consistent with these findings, where 94.9% of our study population had *KRAS* mutations, most frequently in codon 12 (84%), predominantly *KRAS* G12D and G12V (50% and 34%, respectively). Additionally, patients with *KRAS* mutations had a shorter ORR. In addition, our data also revealed that *EPHA7* mutations were significantly correlated to DCR, and patients with *EPHA7* alterations had a worse OS and undesirable PFS. Combined with a recent study suggesting that *EPHA7* mutations could serve as a potential predictive biomarker for immune checkpoint inhibitors in multiple cancers (32), our results suggest that *EPHA7* mutation indicate a poor prognosis, and the use of immune checkpoint inhibitors in *EPHA7*-mutated pancreatic cancer patients may result in better clinical outcomes. *HMGA2* acts as an independent prognostic marker associated with lymph node metastasis (33) and a significantly shorter OS in PDAC (34). Consistently, our results revealed that *HMGA2* mutations were significantly correlated to DCR, and patients with *HMGA2* alterations had an undesirable PFS. Studies have classified PDAC into classical, quasimesenchymal, and exocrine-like subtypes. Classical PDAC showed that higher *GATA6* mRNA expression is associated with a better prognosis (35, 36). Additionally, low *GATA6* expression is associated with shorter OS (37). Our data showed that *GATA6* mutations were associated with a shorter PFS. Other prognostic factors detected in this study, such as *TP53*, *RNF43*, *FGFBR2, FGF23, SMARCA4, CARD11, ADGRA2, CCNE1, ACVR2A*, *NOTCH1*, and *TGFR2*, have not been associated with the prognosis of pancreatic cancer in other studies. A larger sample size is needed for further analysis.

In conclusion, this study used NGS to comprehensively analyze the mutation landscape of pancreatic cancer patients, including primary tumors and metastatic tumors, and the involved signaling pathways. The most commonly mutated genes were *KRAS*, *TP53*, *CDKN2A* and *SMAD4*. In addition, *CDKN2B*, *FAT3*, *MTAP*, and *SMAD4* may be molecular markers that distinguish primary tumors from metastases. Furthermore, *KRAS*, *EPHA7, TP53, HMGA2, GATA6, RNF43*, *FGFBR2, FGF23, SMARCA4, CARD11, ACVR2A*, *NOTCH1*, and *TGFR2* may serve as potential prognostic biomarkers for pancreatic cancer patients who received AG chemotherapy for first-line treatment. Particularly*, KRAS* and *EPHA7* are highly likely to serve as prognostic factors and therapeutic targets in the management of locally advanced pancreatic cancer.

## Data Availability Statement

The original contributions presented in the study are included in the article/supplementary material. Further inquiries can be directed to the corresponding author.

## Ethics Statement

The studies involving human participants were reviewed and approved by The ethics committee of Nanjing Drum Tower Hospital. The patients/participants provided their written informed consent to participate in this study. Written informed consent was obtained from the individual(s) for the publication of any potentially identifiable images or data included in this article.

## Author Contributions

Conception/Design: JD and BL. Provision of study material or patients: JD, XQ, CL, YZ and WK. Collection and/or assembly of data: MT, JC, QL, AL, JH, QG, LW and YQ. Data analysis and interpretation: XZ. Manuscript writing: JD and MX. Final approval of manuscript: JD and BL. All authors have read and approved the submitted version of the manuscript.

## Funding

National Natural Science Foundation of China (No: 82072926), Natural Science Foundation of Jiangsu Province (No: BK20191114), Chen Xiaoping Foundation for the Development of Science and Technology of Hubei Province (No. CXPJJH11900001-2019101), Nanjing Medical Science and technology development project (No: YKK20080)

## Conflict of Interest

Author MX and XZ were employed by Shanghai OrigiMed Co, Ltd, Shanghai, China.

The remaining authors declare that the research was conducted in the absence of any commercial or financial relationships that could be construed as a potential conflict of interest.

## Publisher’s Note

All claims expressed in this article are solely those of the authors and do not necessarily represent those of their affiliated organizations, or those of the publisher, the editors and the reviewers. Any product that may be evaluated in this article, or claim that may be made by its manufacturer, is not guaranteed or endorsed by the publisher.
